# Scratching the surface: native mass spectrometry of peripheral membrane protein complexes

**DOI:** 10.1042/BST20190787

**Published:** 2020-03-04

**Authors:** Cagla Sahin, Deseree J. Reid, Michael T. Marty, Michael Landreh

**Affiliations:** 1 Department of Microbiology, Tumor and Cell Biology, Karolinska Institutet – Biomedicum, Solnavägen 9, 17165 Solna, Sweden; 2 Department of Biology, University of Copenhagen, Ole Maaløes vej 5, 2200 Copenhagen N, Denmark; 3 Department of Chemistry and Biochemistry, University of Arizona, Marvel Hall 542, Tucson, AZ 85721, U.S.A; 4 Bio5 Institute, University of Arizona, Marvel Hall 542, Tucson, AZ 85721, U.S.A

**Keywords:** lipid nanodiscs, membrane protein structure, protein–lipid interactions, structural proteomics

## Abstract

A growing number of integral membrane proteins have been shown to tune their activity by selectively interacting with specific lipids. The ability to regulate biological functions via lipid interactions extends to the diverse group of proteins that associate only peripherally with the lipid bilayer. However, the structural basis of these interactions remains challenging to study due to their transient and promiscuous nature. Recently, native mass spectrometry has come into focus as a new tool to investigate lipid interactions in membrane proteins. Here, we outline how the native MS strategies developed for integral membrane proteins can be applied to generate insights into the structure and function of peripheral membrane proteins. Specifically, native MS studies of proteins in complex with detergent-solubilized lipids, bound to lipid nanodiscs, and released from native-like lipid vesicles all shed new light on the role of lipid interactions. The unique ability of native MS to capture and interrogate protein–protein, protein–ligand, and protein–lipid interactions opens exciting new avenues for the study of peripheral membrane protein biology.

## Peripheral membrane proteins and their interactions

Lipid membranes provide a multitude of crucial functions for the cell. Most importantly, they enable the existence of segregated, controlled chemical milieus by acting as a physical barrier to the outside and between subcellular compartments. They also control the passage of nutrients, cellular components, and signals, facilitated by a myriad of membrane-associated proteins. This membrane proteome can be divided into integral membrane proteins, which contain multiple membrane-spanning segments and extend into both cytoplasm and extracellular space, and peripheral membrane proteins, which insert only partially into the hydrophobic core of the membrane, or associate with the lipid head-groups at its surface, but remain largely in the hydrophilic environment [1]. Although integral membrane proteins transport molecules and signals across the barrier, peripheral membrane proteins often bridge the gap between membrane and processes in the cytosol [2]. They can recruit or transport components to and from the membrane, perform reactions that involve cytosolic and membrane-associated substrates, or transmit signals from the membrane to the nucleus. Additionally, some proteins can alter membrane structures, for example antimicrobial peptides and toxins. In line with such broad involvement in cellular processes, ∼10% of the membrane-associated drug targets are peripheral membrane proteins [3,4].

**Figure 1.**
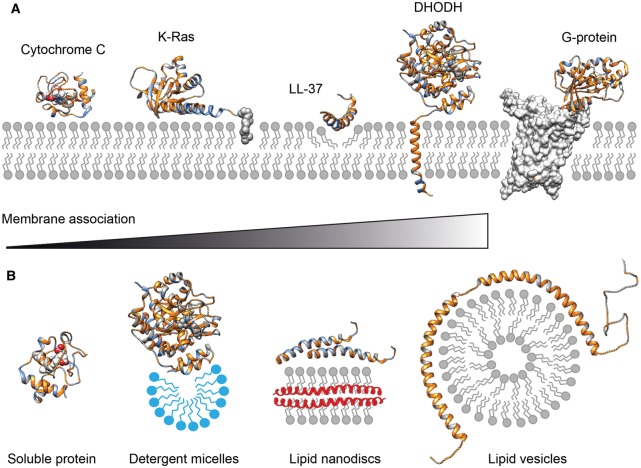
Membrane interactions of peripheral membrane proteins and nMS-compatible vehicles. (**A**) Peripheral membrane protein can associate with the membrane in a variety of ways. Soluble proteins can bind to the membrane via charge and hydrophobic interactions, as exemplified by cytochrome C (PDB ID 1J3S). Additionally, covalent lipid anchors, for example on the C-terminus of K-Ras, can induce a more permanent membrane association (PDB ID 5TAR). The membrane-active peptide LL-37 (PDB ID 2K6O) binds to negatively charged phospholipid head-groups before inserting hydrophobic sidechains into the membrane core to induce pore formation. The mitochondrial enzyme DHODH (PDB ID 2PRM) has an N-terminal transmembrane helix to ensure correct orientation of the substrate binding site towards the membrane. G-proteins (PDB ID 5G53) are not membrane-associated themselves but require individual phospholipids such as PIP2 to form complexes with their membrane-inserted GPCR targets. Hydrophobic regions are rendered in gold, positively charged positions are shown in blue. (**B**) Strategies to stabilize peripheral membrane proteins for nMS. Although soluble proteins such as cytochrome *c* can be analyzed by nMS from aqueous solutions, membrane-associated DHODH requires detergent micelles (blue) for stability. Lipid nanodiscs can be used to preserve protein interactions that form on the membrane surface, for example in LL-37 oligomerization. Extensive membrane interactions can be studied using lipid vesicles that provide a large surface for interactions, for example with the amphipathic helices of α-synuclein (PDB ID 2KKW).

As our understanding of the nature of the cell membrane evolves, it has become clear that it is not composed of homogeneously distributed lipids and proteins. In fact, the membrane is highly organized, with specific lipids and proteins forming functional units that allow for spatial and temporal regulation of their activities [5,6]. A growing body of evidence suggests that specific lipid interactions control the structures and functions of many integral membrane proteins, including their oligomerization, activity, and stability [7–9]. In some cases, X-ray crystallography and cryo-electron microscopy offer detailed insights into lipid binding [10,11]. It is therefore not surprising that lipid-mediated regulation extends to the family of peripheral membrane proteins. In line with their broad range of functions, the mechanisms of membrane binding are equally diverse, involving hydrophobic association, covalent lipid anchors, and recognition of specific lipid species or properties (Figure 1A). The functions of many peripheral membrane proteins are therefore crucially dependent on interactions with the lipids that constitute their target membranes, which in turn control the specificity of association, (sub)cellular localization, and effects on protein and membrane structures.

The impact of lipid interactions on the biological roles of peripheral membrane proteins is illustrated by their role in health and disease. A striking example is the small GTPase K-Ras, which normally relays proliferation signals by recruiting and activating growth factors but is mutated in up to 20% of all human cancers [12]. K-Ras is associated with the plasma membrane via a farnesyl anchor, and consequently, this modification has been targeted to disrupt the aberrant activity of oncogenic K-Ras mutants [13]. However, the protein contains a hypervariable C-terminal extension that mediates lipid-specific protein orientation on the membrane, which compensates for loss of the farnesyl-mediated membrane association and complicates pharmacological intervention [14,15].

Although the native membrane interactions of structured proteins like K-Ras are themselves not detrimental, membrane associations of disordered proteins such as huntingtin, α-synuclein, and amyloid-β have been implicated in the number of human neurodegenerative disorders. Here, contacts with the surface of a lipid bilayer induce the formation of amphipathic helices in different segments, leading to local accumulation, uncontrolled aggregation, and membrane damage believed to contribute to disease pathology. Notably, one of these proteins, α-synuclein, is a peripheral membrane protein that shares sequence features with apolipoproteins and associates with synaptic vesicles *in vivo* and negatively charged phospholipids *in vitro* [16]. Several naturally occurring mutations have been found to alter lipid-binding preferences of α-synuclein, potentially influencing its propensity to form toxic aggregates on or off the membrane [16–18]. Interestingly, the role of lipids in the aggregation of these proteins shares similarities with membrane interactions found in antimicrobial peptides (AMPs) [19].

## Native mass spectrometry of membrane proteins

Despite their biological importance, many aspects of the interactions between peripheral membrane proteins and lipids are only partially understood. This is due to the complex chemical nature of the cell membrane, as well as the diversity of lipid–protein interactions that range from fleeting, non-specific associations to the formation of highly specific complexes. The need to understand how lipids shape structure and function of peripheral membrane proteins has led to the development of a number of tools for their biophysical characterization, including NMR [20], which allows structure determination of proteins bound to detergent micelles; EPR [21], which informs about binding geometries on bilayers; and hydrogen-deuterium exchange MS [22], which maps lipid-binding regions and local conformational changes. Molecular dynamics (MD) simulations are routinely employed to study protein–lipid interactions and provide the highest level of detail with regard to their structures and dynamics [23,24].

A relatively new addition to this toolbox is native mass spectrometry (nMS). In nMS, protein complexes are ionized directly from non-denaturing solutions using nano-electrospray ionization (nESI) without distorting their native interactions [25]. The desolvated complexes with intact non-covalent interactions are then transferred into the vacuum region of a mass spectrometer for molecular mass measurements. In addition, the complexes can be dissociated through collisions with buffer gas molecules inside the mass spectrometer to release subunits or ligands, providing information about their components and relative stabilities. In this manner, it is possible to monitor protein oligomerization, determine ligand preferences, and delineate subunit connectivities [26].

Importantly, the same approach can be applied to integral membrane proteins that need to be embedded in a highly amphiphilic environment to retain their native structure and interactions in solution [27–29]. Robinson and co-workers demonstrated that protective vehicles such as detergent micelles can be used to facilitate the analysis of intact membrane protein complexes by nMS [30,31]. Here, the protein complex is desolvated and ionized together with the surrounding detergent micelle. Subsequent high-energy collisions with buffer gas in the ion source or ion trap region of the mass spectrometer dissociate the bound detergent molecules, leaving the intact protein complex for mass analysis. Several studies have since then significantly extended the range of MS-compatible vehicles, adapting different strategies to stabilize membrane proteins in native-like environments that can be removed during nMS analysis. As a result, nMS has proven particularly useful for the analysis of protein–lipid interactions, revealing the roles of specifically bound lipids in membrane protein folding, stabilization, and oligomerization.

Specifically, three major strategies have been developed (Figure 1B) [32]. The first and most common approach is protein solubilization, where the protective membrane environment is replaced by a detergent micelle or an amphiphilic polymer [30,33]. This method relies on the removal of most, or all, membrane lipids, often leaving ‘naked’ membrane proteins that may have lost structurally or functionally important lipid ligands. The second approach involves lipid nanodiscs, where the protein is embedded in an artificial membrane [34,35]. Here, proteins are extracted from the membrane with detergent but reconstituted into small, flat bilayers held together by a scaffolding protein. This method utilizes defined lipid compositions to mimic different membranes. Most recently, a third approach using membrane vesicles was demonstrated, where the planar membrane is broken up to yield nESI-compatible fragments containing the proteins of interest. These can be produced by sonication of artificial lipid mixtures or native cell membranes, yielding vesicles of varying sizes that can be desolvated for nMS analysis [36]. Here, we will outline how each of these strategies can be adopted to enable nMS of peripheral membrane protein complexes.

## Solubilizing peripheral membrane proteins for nMS

There are different modes of membrane association across the diverse family of peripheral membrane proteins (Figure 1A). Some cytosolic proteins bind only transiently to the membrane, and do not expose larger hydrophobic areas that would reduce their stability in solution. In these cases, the folded proteins and their interactions are easily amenable to nMS analysis in aqueous buffers due to their good solubility.

A prominent example is cytochrome *c*, which binds specifically to cardiolipin (CDL) in the inner mitochondrial membrane and acts as an electron transporter between the membrane-embedded respiratory chain complexes III and IV. No stable protein–lipid complexes could be detected directly by nMS of cytochrome *c* in the presence of CDL [37]. However, nMS in combination with ion mobility measurements revealed that the protein adopts a more compact conformation in the gas phase when the lipid is present, which likely arises from interactions between the protein and CDL clusters in the solution that are disrupted during desolvation [37].

Other soluble proteins are more closely associated with the membrane, like K-Ras, which is stable in solution but remains anchored to the membrane via palmitoylated cysteine residue. To facilitate nMS analysis, Laganowsky and co-workers therefore used a C-terminally truncated version lacking the last 19 amino acids including the farnesylation site at Cys180 [38]. With this modification, the authors were able to preserve the interactions between K-Ras and nucleotides under native MS conditions. By monitoring the conversion of GTP to GDP in the intact protein complex, they could determine kinetic parameters for GTP hydrolysis and map the impact of oncogenic mutations on the GTPase activity of K-Ras [38].

The degree of membrane association is increased even further in a number of peripheral membrane receptors and enzymes, which brings about the complication of having to stabilize the proteins in solution. These proteins contain single transmembrane helices that bring folded, soluble domains close to the membrane, and engage in interactions in the membrane as well as in the cytosol or the extracellular space. Because only some parts are membrane-inserted, protein engineering strategies can be employed to generate soluble variants. For example, the antigen-presenting Cluster of Differentiation 1b receptor (CD1b) binds and exposes a lipid molecule from its host membrane to inform T-lymphocytes about alterations in the cell's lipid metabolism [39]. The lipid binding site is located in the extracellular soluble domain, and lipid loading occurs during translation in the endoplasmic reticulum of the cell. Indeed, the deletion of the C-terminal transmembrane helix resulted in a soluble, folded lipid-binding protein [40]. nMS analysis of the truncated protein extracted from mouse cells revealed loading with endogenous phospholipids and a strong preference for phosphatidylcholine.

Yet, strategies to turn peripheral membrane proteins into soluble proteins may not always be feasible. The enzyme dihydroorotate dehydrogenase (DHODH) catalyzes the oxidation of dihydroorotate to orotate, using a bound flavin mononucleotide (FMN) as cofactor and ubiquinone in the mitochondrial membrane as an electron acceptor. Like CD1b, DHODH contains a single N-terminal transmembrane helix, but it requires detergent to retain its native fold and display dehydrogenase activity *in vitro*even when the transmembrane region is removed [41]. Crystallographic analysis showed the presence of two amphiphilic helices arranged in a horseshoe shape that forms the entrance to the binding site for the membrane-associated substrate ubiquinone (Q10). In several structures, the entrance is surrounded by detergent molecules, suggesting that this region is reliant on membrane interactions *in vivo* [41–43].

**Figure 2.**
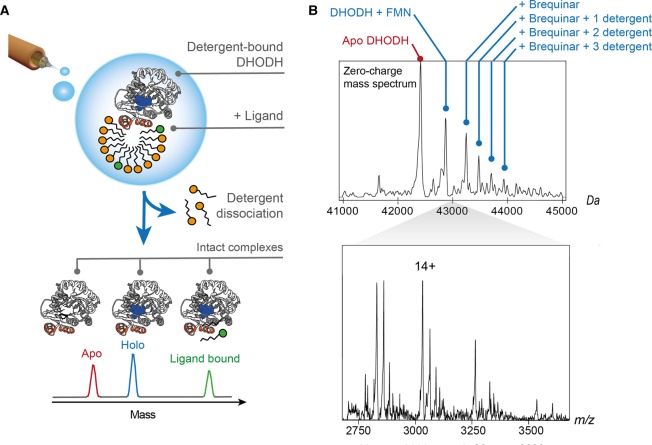
nMS of the peripheral membrane protein DHODH shows concomitant binding of cofactor, inhibitor, and detergent molecules. (**A**) The nMS approach used for detergent-solubilized integral membrane proteins facilitates the analysis of intact DHODH complexes. Release of the desolvated protein from detergent micelles by collisional activation preserves interactions with the FMN cofactor as well as exogenous ligands. (**B**) nESI-MS of DHODH in the presence of detergent and Brequinar shows a series of peaks corresponding to the protein with its FMN cofactor in complex with one Brequinar molecule and zero, one, two, or three detergent molecules. The deconvoluted spectrum is shown above the corresponding mass spectrum. Adapted from reference [44] with permission. Copyright 2018 Elsevier.

To dissect the relationship between DHODH folding, inhibitor binding, and membrane interactions, Costeira-Paulo et al. [44] performed nMS analysis of the protein embedded in detergent micelles that were subsequently stripped away by collisional activation to obtain well-resolved spectra of DHODH with bound FMN cofactor and ligands (Figure 2A). The energies required to release DHODH were lower than those normally used for integral membrane proteins, in line with a more labile protein-micelle interaction. When different mitochondrial lipids were added directly to the electrospray solution, DHODH preferentially forms complexes with CDL, in line with its location near the respiratory complexes in the mitochondrial membrane. Turning to nMS of the interactions between DHODH and Brequinar, an anticancer drug that occupies the membrane-accessible Q10 binding site [45], only the holo-enzyme with its FMN cofactor was able to bind the inhibitor, and the DHODH-inhibitor complex was able to retain up to three detergent molecules. From this, the order of interactions could be delineated: The FMN cofactor at the center of the enzyme is required to form the Q10 binding site that is occupied by Brequinar. Once the inhibitor is bound, the membrane-associated domain is stabilized by detergent molecules that mimic lipid interactions. The observations from nMS were then used to inform all-atom MD simulations of full-length DHODH associated with a phospholipid membrane. The simulations revealed that interactions with negatively charged lipid head groups anchor the entrance to the Q10 binding site to the membrane and likely provide crucial stabilization required for efficient inhibitor binding.

## Use of nanodiscs for the study of peripheral membrane protein interactions

Another approach for nMS analysis of peripheral membrane protein is to use membrane mimetics as soluble lipid bilayers. Lipoprotein nanodiscs are a promising platform because their size, stability, homogeneity, versatility, and relative monodispersity are ideal for nMS. Importantly, the lipid bilayer of nanodiscs more accurately models the biological membrane surface than detergent micelles.

**Figure 3.**
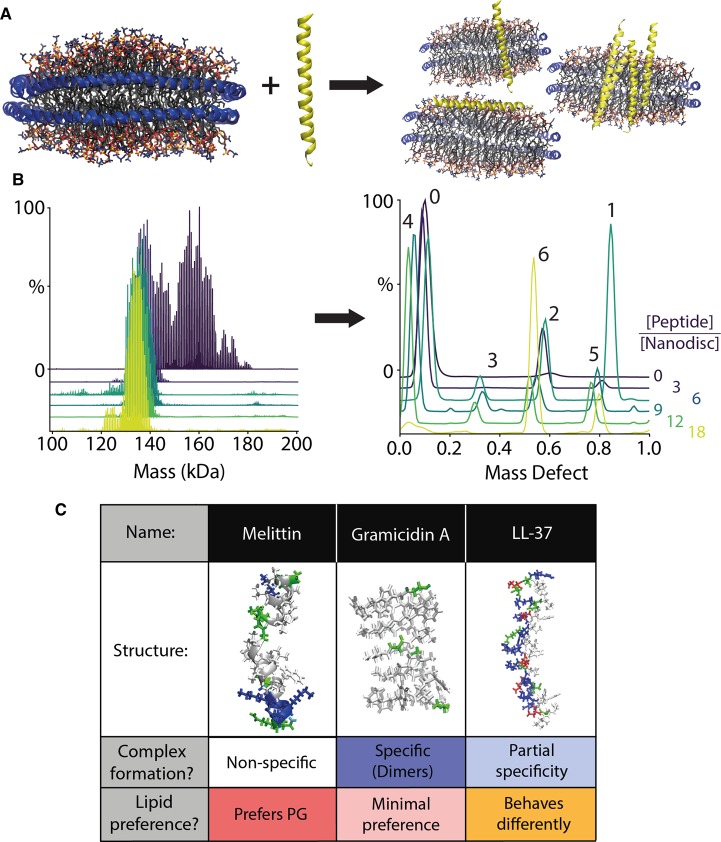
Peripheral membrane protein complexes of AMPs and lipid nanodiscs. (**A**) In a catch-and-hold assay, LL-37 is added to nanodiscs and allowed to interact with the membrane. (**B**) LL-37 nanodisc complexes are then analyzed directly by nMS to determine the intact mass distribution (left). The corresponding mass defects (right) reveal the distribution of peptide stoichiometry of LL-37 molecules per nanodisc (annotated above) as a function of the solution peptide to nanodisc molar ratio, shown in different colors. Adapted with permission from [64]. Copyright 2019 American Chemical Society. (**C**) An overview showing each AMP's structure, stoichiometric specificity, and lipid preference.

Nanodiscs are composed of a lipid bilayer encircled by two amphipathic membrane scaffold proteins (MSPs) [46,47], which are truncated forms of human apolipoprotein AI. Shorting or lengthening the MSP belts enables nanodiscs to be formed with various sizes ranging from ∼6–50 nm in diameter [48,49]. To create nanodiscs, lipids and MSP are mixed in the presence of detergent and allowed to incubate near the phase transition temperature of the lipids. Upon removal of detergent by dialysis or porous hydrophobic beads, a discoidal bilayer domain is formed with two molecules of MSP encircling the hydrophobic regions of each leaflet (Figure 3A). Nanodiscs are then purified by size exclusion chromatography.

The first application of nanodiscs to nMS was by Klassen and co-workers to study binding of peripheral membrane proteins to glycolipids in nanodiscs [50]. The authors studied binding of the B subunit of cholera toxin (CTB) to glycosphingolipid (GSL) receptors incorporated into 1,2-dimyristoyl-sn-glycero-3-phosphocholine (DMPC) nanodiscs. CTB is a homopentamer containing carbohydrate binding sites that exhibit high affinity for GM1, the target GSL of CTB. Using a ‘catch-and-release' technique, nanodiscs containing GM1 were incubated with CTB, allowing nanodiscs to ‘catch' bound CTB. The resulting CTB-nanodisc complexes were then directly ionized by nESI. Inside the mass spectrometer, collisions with buffer gas were used to ‘release' CTB from the nanodiscs. Caught GM1 remained bound to CTB during ejection, and thus, binding could be quantified directly by examining the relative intensities of ejected CTB + GM1. Building from this initial method, nanodiscs were then made from lipid mixtures extracted from a human epithelial cell line known to contain GM1, which enabled investigation of the interactions of the protein-GSL complexes in an environment that more closely mimicked *in vivo* conditions [50]. Additional research from Klassen and co-workers has improved quantitation with the method [51], extended these approaches to Saposin A-based picodiscs [52], and employed catch-and-release assays for the investigation of various carbohydrate-binding proteins with a panel of glycolipids [53,54].

Similarly, the first applications of nMS to integral membrane proteins in nanodiscs by Hopper et al. [34] used collisional activation to release membrane proteins from the nanodiscs. Marty et al. [35] showed further that large numbers of lipids bound to the ejected proteins could still be detected, providing vital information about interactions within the lipid environment. In these and other studies, integral membrane proteins were co-assembled into nanodiscs and then released in the mass spectrometer, a ‘build-and-release' approach [50,55–57].

However, the monodispersity and homogeneity of nanodiscs allows them to be analyzed by nMS without collisional activation, which better preserves native interactions. Marty et al. first demonstrated that empty nanodiscs composed of a variety of lipids and without embedded membrane proteins can be preserved during nMS [58–62]. Recently, Keener et al. [63] discovered that nanodiscs with embedded integral membrane proteins could be preserved and resolved by nMS without the need for ejecting the membrane protein]. Using chemical additives that modulate the number of charges acquired during nESI, the intact integral membrane protein-nanodisc complex was preserved for mass analysis. This ‘build-and-hold' approach avoids potential distortions from dissociation of the nanodiscs inside the mass spectrometer and allows the oligomeric state of membrane proteins to be measured within an intact lipid bilayer.

Building on the progress with integral membrane proteins, Walker et al. [64] discovered that nMS can be powerful for measuring the stoichiometry of lipid bilayer-associated AMP complexes added directly to intact nanodiscs. With this ‘catch-and-hold' approach, the membrane protein/peptide of interest does not need to be ejected from the nanodiscs to measure the oligomeric state but is measured directly from the mass of the intact nanodisc complexes. In this example, three different AMPs were added to nanodiscs containing either DMPC or 1,2-dimyristoyl-sn-glycero-3-phospho-(1′-rac-glycerol) (DMPG), which model mammalian and bacterial membranes, respectively. The AMPs were titrated into preformed nanodiscs and allowed to directly insert into the nanodisc membrane, mimicking how they interact with natural membranes. The resulting peptide-nanodisc complexes were measured directly via nMS. Each AMP exhibited unique stoichiometries, which varied depending on the lipid type and AMP concentration (Figure 3).

One noteworthy example was LL-37, a 37-residue human cathelicidin that is believed to form oligomers in the presence of the lipid bilayer [65]. When added to DMPC nanodiscs, LL-37 tends to be destabilizing, in agreement with prior results [66]. However, upon titration of LL-37 into DMPG nanodiscs, LL-37 formed oligomeric complexes within the lipid bilayer that showed partial specificity for stoichiometries of 2, 4, 5, and 6 peptides per nanodisc. Furthermore, increased collisional activation of DMPG nanodisc complexes with high molar ratios of LL-37 (a catch-and-release approach) resulted in ejection of oligomers that retained some bound DMPG, which confirmed the presence of oligomeric complexes within the nanodisc membrane. In comparison, gramicidin A formed dimers in both DMPC and DMPG nanodiscs. Melittin showed greater incorporation in DMPG yet did not display any specific oligomeric complexes. Interestingly, nMS of LL-37 in detergent micelles revealed lipid preferences, but did not capture oligomerization [67]. These results therefore show that nMS of intact nanodiscs can be used to measure the stoichiometry of AMP complexes associated with lipid bilayers.

One challenge of the catch-and-hold approach is that it may be difficult to hold on to peripheral membrane proteins during nMS. In other words, peripheral membrane proteins like CTB that interact through only a few bound lipids may be easily released in the gas phase even under very gentle ionization and analysis conditions. Promising new charge reducing reagents may be helpful to address this challenge by stabilizing fragile nanodisc complexes in the gas phase [68,69]. Another challenge is that the mass distribution can become very complex and eventually uninterpretable as different types of lipids and peptide/proteins are added to the nanodisc complex. This challenge can be partially addressed through careful experiment design, and the use of lipids with similar or resonant masses helps simplify the mass distributions [60,61]. In any case, further research will be necessary to establish the limits of the technique. AMPs have been suggested to act according to the carpet, toroidal pore, or barrel-stave models, which may rely on different oligomerization propensities. Therefore, the stoichiometries observed by native MS have the potential to shed light on the mechanisms of AMP toxicity.

As discussed in the previous examples, nanodisc-based nMS using both catch-and-release and catch-and-hold techniques provides unique information about peripheral membrane proteins and their interactions with and within the lipid bilayer. Nanodiscs are currently the only membrane mimetic that can be maintained intact for native ESI-MS analysis. Other mimetics without lipoprotein belts, including bicelles, amphipols, SMALPs, liposomes, and micelles, likely have too much mass polydispersity to be resolved by native MS without breaking the complex apart to some degree. However, membrane mimetics with other protein belts, such as Saposin A, [50,59] or with peptide belts [70] may be amenable to intact analysis because they limit the mass polydispersity introduced by small molecules or polymers. But, these complexes are less stable than conventional MSP nanodiscs and have not yet been resolved intact with proteins bound. The unique ability of nanodiscs to be captured intact, coupled with their customizability, affords a broad range of biochemical applications and has expanded the repertoire of approaches that can be used to investigate peripheral membrane protein oligomerization and lipid interactions. For example, nanodiscs have been successfully integrated with HDX-MS [71,72]. In this context, the combination of nMS with top-down sequencing methods offers a chance to connect lipid binding with structural changes.

## Lipid vesicles preserve specific protein interactions in the gas phase

The most recent development in nMS of integral membrane proteins is the use of vesicles derived from intact phospholipid membranes [36]. Large membranous structures such as extracellular vesicles or disrupted bacterial membranes are broken up by high-energy sonication to form smaller particles that are amenable to nESI. Membrane proteins remain embedded in these particles and are subsequently released by high-energy collisions inside the mass spectrometer analogous to the release of proteins from detergent micelles. Importantly, the proteins are thus discharged from a vehicle that essentially corresponds to their native lipid environment. In contrast, detergents can affect protein–lipid interactions [73].

It is noteworthy that the earliest nMS studies on any membrane-associated protein utilized a similar approach to investigate lipid interactions with apolipoprotein (apo-)CII [74,75]. To demonstrate the feasibility of using lipid vesicles in nMS, Hansen et al. showed in their 2005 study that vesicle fragments of up to 95 lipid molecules could be preserved in the gas phase. nMS of vesicles with apo-CII under gentle desolvation conditions resulted in well-resolved spectra showing complexes with up to 22 lipid molecules, more than expected based on the number of available binding sites. These results indicated that apo-CII could interact with lipid clusters, and thus underscored the advantage of using intact lipid vesicles to study membrane interactions. Interestingly, nMS of vesicles composed of multiple lipid species showed non-random assemblies and self-sorting based on differences in lipid chemistry. In the presence of apo-CII, however, these distributions were not reflected in the number and types of protein-bound lipids. Instead, apo-CII exhibited preferential lipid interactions leading to changes in lipid vesicle composition.

More recently, a similar strategy was adopted to study lipid preferences of the N-terminal amphipathic helix of the huntingtin protein, aggregation of which is associated with amyotrophic lateral sclerosis. Valentine and co-workers used nMS to monitor the interactions of a peptide encompassing huntingtin residues 1–17 with lipid vesicles composed of different zwitterionic lipids [76]. By comparing the signal intensities and stoichiometries of the resulting complexes, the authors were able to show that peptide oligomerization is enhanced by the presence of vesicles, and preferential binding of doubly unsaturated lipids. MD simulations suggested that the peptide more readily inserts into bilayers composed of these lipids as they are less densely packed, giving rise to both charge-based and hydrophobic interactions.

In the cases of huntingtin and apo-CII, the associations with lipid vesicles could easily be disrupted during nESI. Considering the observation that DHODH requires less energy for release from detergent than an integral membrane protein, we conclude that such low-stability associations appear to be a common feature among peripheral membrane proteins. However, another protein system required a significantly different approach. The antimicrobial peptide gramicidin A attaches to the membrane from both sides of the bilayer and in this manner forms membrane-spanning dimers that act as ion-transmitting pores, leading to cell death. The peptide is thus converted from a peripheral to an integral membrane protein in the process [77]. To facilitate analysis of the intramembrane dimer, Russell and co-workers developed a nMS strategy where the peptide is released from phospholipid vesicles by freeze-drying and resuspension in hydrophobic solvent. Although lipid interactions are lost in this way, the conformations of the labile dimer can be preserved [78]. Using nMS in combination with ion mobility, the authors could show that the conformation of the gramicidin A dimer is modulated by the cholesterol content of the vesicle, and may thus affect the specificity of its antimicrobial effect [79].

## Perspective

- Although just over a decade old, the field of membrane protein nMS has already provided a host of novel biological insights. The rapid progress has been enabled by a series of significant technical innovations that have made possible the analyses of increasingly complex membrane protein systems, and particularly the elusive role of membrane protein–lipid interactions in health and disease.
- We outline here that most of the nMS strategies tailored for integral membrane proteins can be applied with no, or only minor, modifications to the study of peripheral membrane proteins. As a result, mass spectrometric analysis of intact peripheral membrane protein complexes is now possible, capturing their interactions with detergent micelles [44], lipid nanodiscs [64], lipid vesicles [74], and even integral membrane protein partners [80].
- Based on these developments, we predict that nMS will enable new insights into elusive lipid–protein interactions. nMS has the potential to continue to make significant contributions to poorly understood disease mechanisms such as the membrane-mediated formation of protein aggregates in neurodegeneration [75], the mechanism of action of novel antimicrobial peptides [76], and lipid-encoded signaling in cancer [77].
- nMS can be integrated with other structural biology methods to yield detailed molecular models [81]. For peripheral membrane proteins, this includes validation of insights from MD simulations, and resolving interactions not accessible by X-ray crystallography.

## Open Access

Open access for this article was enabled by the participation of the Karolinska Institute in an all-inclusive *Read & Publish* pilot with Portland Press and the Biochemical Society.

## Abbreviations

AMP: antimicrobial peptide
CD1b: cluster of differentiation receptor 1b
CDL: cardiolipin
CTB: Cholera toxin B
DHODH: dihydroorotate dehydrogenase
DMPC: 1,2-dimyristoyl-sn-glycero-3-phosphocholine
DMPG: 1,2-dimyristoyl-sn-glycero-3-phospho-(1′-rac-glycerol)
FMN: flavin mononucleotide
GSL: glycosphingolipid
MD: molecular dynamics
nESI: nano-electrospray ionization
nMS: native mass spectrometry
Q10: ubiquinone
